# Influence of Age, Past Smoking, and Disease Severity on TLR2, Neutrophilic Inflammation, and MMP-9 Levels in COPD

**DOI:** 10.1155/2013/462934

**Published:** 2013-03-31

**Authors:** Jodie L. Simpson, Vanessa M. McDonald, Katherine J. Baines, Kevin M. Oreo, Fang Wang, Philip M. Hansbro, Peter G. Gibson

**Affiliations:** ^1^NHMRC Centre for Respiratory and Sleep Medicine, Faculty of Health, School of Medicine and Public Health, The University of Newcastle, Callaghan, NSW 2308, Australia; ^2^Department of Respiratory and Sleep Medicine, Hunter Medical Research Institute, Newcastle, NSW 2305, Australia; ^3^Priority Research Centre for Asthma and Respiratory Diseases and Hunter Medical Research Institute, The University of Newcastle, Callaghan, NSW 2308, Australia; ^4^School of Nursing and Midwifery, The University of Newcastle, Callaghan, NSW 2308, Australia; ^5^Norman Bethune Medical College, Ji Lin University, Changchun 130021, China

## Abstract

Chronic obstructive pulmonary disease (COPD) is a common and serious respiratory disease, particularly in older individuals, characterised by fixed airway obstruction and persistent airway neutrophilia. The mechanisms that lead to these features are not well established. We investigated the contribution of age, prior smoking, and fixed airflow obstruction on sputum neutrophils, TLR2 expression, and markers of neutrophilic inflammation. Induced sputum from adults with COPD (*n* = 69) and healthy controls (*n* = 51) was examined. A sputum portion was dispersed, total, differential cell count and viability recorded, and supernatant assayed for CXCL8, matrix metalloproteinase- (MMP-) 9, neutrophil elastase, and soluble TLR2. Peripheral blood cells (*n* = 7) were stimulated and TLR2 activation examined. TLR2 levels were increased with ageing, while sputum neutrophils and total sputum MMP-9 levels increased with age, previous smoking, and COPD. In multivariate regression, TLR2 gene expression and MMP-9 levels were significant independent contributors to the proportion of sputum neutrophils after adjustment for age, prior smoking, and the presence of airflow obstruction. TLR2 stimulation led to enhanced release of MMP-9 from peripheral blood granulocytes. TLR2 stimulation activates neutrophils for MMP-9 release. Efforts to understand the mechanisms of TLR2 signalling and subsequent MMP-9 production in COPD may assist in understanding neutrophilic inflammation in COPD.

## 1. Introduction

Chronic obstructive pulmonary disease (COPD) is a disease of global significance that is a major contributor to mortality and morbidity in older people [1]. Cigarette smoking is a major risk factor, 64 million people are affected worldwide, and it is the 4th most common cause of death [2]. COPD is characterized by airflow obstruction that is progressive and incompletely reversed by current therapy [3]. This underscores the need to better understand the disease mechanisms, to facilitate the search for new treatment modalities in COPD [4]. Airway inflammation is a key element of COPD and typically involves persistent neutrophilic airway inflammation (neutrophilic bronchitis). Neutrophilic bronchitis is persistent and not reversed after the removal of stimuli such as tobacco smoke and may contribute to disease progression. Understanding the mechanisms of persistent neutrophilia is likely to inform the identification of new therapeutic targets in COPD.

We previously reported the involvement of the innate immune system in airway diseases that are characterized by neutrophilic bronchitis (the neutrophilic subtype of asthma and bronchiectasis) [5]. TLR2 gene expression is elevated in patients with COPD [6], and surface expression is reduced in airway cells from patients with COPD compared to controls [7, 8] suggesting a disease-related alteration in TLR2 expression. Ageing is also associated with airway neutrophilia implicating a role for altered innate immune responses in older individuals. It is not clear if the changes in neutrophil accumulation with advancing age are accompanied by enhanced CXCL8 and neutrophil protease (e.g., NE) release. Smoking also induces and enhances neutrophil responses. Nevertheless, the extent to which factors such as age and past smoking impact on the persistent neutrophilic bronchitis in COPD is uncertain.

Here we report on our investigations of these issues. We hypothesized that neutrophilic airway inflammation and TLR2 expression would increase with age and past smoking, but would be further unregulated in COPD. We assessed neutrophilic inflammation and the associated expression of neutrophil and innate immune mediators whilst controlling for the effects of age, smoking, and the presence and severity of airway obstruction. A disease specific dysregulation of these components may represent potential future targets for therapy in patients with COPD.

## 2. Material and Methods

### 2.1. Ethics Statement

Participants gave written informed consent. The Hunter New England Area Health Service and University of Newcastle Research Human Ethics Committees approved this study.

### 2.2. Participant Recruitment

We recruited consenting adults who were greater than 55 years of age with COPD (*n* = 100) from a tertiary care setting of the Respiratory and Sleep Medicine Ambulatory Care Service at John Hunter Hospital, NSW, Australia. A group of healthy controls (*n* = 61,  *n* = 22 < 55 years, and *n* = 39 > 55 years, resp.) were recruited from the community by advertisement. COPD diagnosis was assessed by a physician and by post-bronchodilator spirometry. Participants were excluded (*n* = 16) if the results did not meet GOLD criteria of postbronchodilator FEV_1_/FVC < 70%  and  FEV_1_ < 80%  [3]. 

Participants had no reported exacerbations or alterations in respiratory medications in the previous four weeks and were excluded from analysis if they were taking maintenance oral corticosteroids (*n* = 3) or long-term antibiotic therapy (tetracycline and macrolide, *n* = 2) or had a diagnosis of COPD secondary to a diagnosis of bronchiectasis (*n* = 4). Further exclusion criteria were current smoking (*n* = 6  COPD, *n* = 3 control) or having ceased smoking in the past 6 months (*n* = 1 control). Healthy controls had normal lung function and no diagnosis of airways disease. Healthy controls were excluded if they reported respiratory symptoms in combination with one or more of either airflow obstruction, airway inflammation, or airways hyperresponsiveness to hypertonic saline (*n* = 6). The final group for analysis consisted of 69 patients with COPD and 51 healthy controls. 

### 2.3. Design

Participants attended three visits. Pre- and post-bronchodilator spirometry, symptoms, quality of life, medication use, smoking status, and exhaled carbon monoxide were assessed at visit one, and sputum induction was undertaken. At visit two, skin allergy tests and a repeat sputum induction (if required) were performed. On the final visit a single breath diffusing capacity test was performed. Sputum was processed for inflammatory cell counts, gene expression and protein levels of inflammatory mediators, and bacteria analysis. A group of 7 participants with COPD provided an additional sample of peripheral blood for isolation and stimulation of granulocytes and mononuclear cells to investigate the activation of TLR2 *in vitro*.

### 2.4. Quality of Life and Health Care Utilisation

Quality of life was examined using the SF-36 questionnaire [9]. We assessed health care utilisation by asking participants about their visits to hospital, emergency room, and General Practitioner, due to their chest disease. The number of courses of oral corticosteroids or antibiotics for their disease in the past 12 months was also recorded.

### 2.5. Mucus Production and Chronic Bronchitis

Mucus production was noted as positive if the participant reported an affirmative answer to the following questions: “Do you cough and produce sputum/phlegm?”, “Do you usually bring up phlegm from your chest?”, or “Do you usually have phlegm in your chest that is difficult to bring up when you don't have a cold?” The presence of chronic bronchitis was assessed using questions taken from the 1978 ATS/DLD Respiratory Symptom Questionnaire [10].

### 2.6. Smoking Assessment

A smoking history was taken and smoking pack-years determined. Participants underwent exhaled carbon monoxide (eCO) measurements, determined by electrochemical detection with a Smokerlyzer (Bedfont, Kent, UK; detection limit of 1 ppb). All included participants had an eCO of less than 10 ppm confirming their nonsmoking status [11]. 

### 2.7. Pulmonary Function Tests

Participants withheld bronchodilators for their duration of action before testing. Three reproducible measurements of FEV_1_ and forced vital capacity (FVC) were obtained (KoKo PD Instrumentation, USA) before and after inhalation of 200 *μ*g salbutamol via a metered dose inhaler with valved holding chamber (Volumatic, Allen and Hanburys, Australia). These measures were compared with predicted values according to Knudson et al. [12]. The carbon monoxide transfer coefficient (KCO) was determined according to ATS guidelines (MedGraphics Elite DX, Medical Graphics Corporation, USA) [13].

### 2.8. Sputum Induction and Analysis

Sputum induction with hypertonic saline (4.5%) was performed as previously described [14]. Selected sputum (100 *μ*L) was either stored in buffer RLT (Qiagen, Hilden, Germany) at −80°C for RNA extraction or dispersed using dithiothreitol (DTT). A total cell count of leukocytes and viability was performed on filtered suspensions. Following centrifugation, supernatant was stored at −80°C for the assessment of CXCL8, total MMP-9, and NE. When sputum volume was <100 *μ*L, a cell smear was prepared, and total cell count and viability data were not collected. Cytospins were prepared and stained (May-Grunwald Giemsa), and a differential cell count was obtained from 400 nonsquamous cells.

### 2.9. Sputum Gene Expression

RNA was extracted from induced sputum samples using RNeasy Mini Kit (Qiagen, Hilden, Germany) and quantitated using Quant-iT RiboGreen RNA Quantitation Assay Kit (Molecular Probes Inc., Invitrogen, Eugene, OR, USA). 

Gene expression for TLR2, TLR4 was analysed using quantitative real-time PCR. Sputum RNA (200 ng) was reverse transcribed to cDNA and combined with TaqMan primers and probes for TLR2 or TLR4 and the reference housekeeping gene eukaryotic 18S ribosomal RNA in duplex real-time PCRs, as previously described (7500 Real Time PCR System, Applied Biosystems, USA) [15]. Relative gene expression to 18S RNA and an internal calibrator was determined using the  2^−ΔΔ Ct^ method.

### 2.10. Sputum Supernatant Mediator Assessment

Soluble TLR2, CXCL8, and total MMP-9 were assessed using commercial ELISA kits (R&D Systems, Minneapolis, MN, USA) and NE was measured with the InnoZyme Human Neutrophil Elastase Immunocapture Activity Assay (Calbiochem, Merck, Kilsyth, VIC, Australia).

### 2.11. Peripheral Blood Isolation and Stimulation

Peripheral blood was collected in sodium citrate (18 mL), and granulocytes and mononuclear cell fractions were isolated using density gradient separation (Percoll GE Healthcare, Rydalmere, NSW, Australia). Isolated cells were cultured (1 × 10^6^ cells/mL) in RPMI1640 complete (1% FCS, 0.05% penicillin/streptomycin), 0.5% HEPES (Invitrogen, Mulgrave, VIC, Australia) and stimulated with agonists of TLR2 (Pam3CysK4, 1000 ng/mL; TLR2 agonist; EMC Microcollections, Tübingen, Germany). Cell-free supernatants were collected after 24 hours, and cells were analysed using flow cytometry or stored in RLT buffer (Qiagen, Doncaster, VIC, Australia) with 0.01%  *β*-mercaptoethanol (Sigma-Aldrich, Castle Hill, NSW, Australia) for assessment of gene expression. CXCL8, IL-6, IL-1b, TNF-a were measued by flow cytometric bead array (Cytometric Bead Array, BD Biosciences, USA), with data exported from FACSDiva 5 software and analysed using FCAP Array Software (BD Pharmingen, USA). MMP-9 (R&D Systems, USA) and NE (Calbiochem, USA), were measured using ELISA and analysed on FluoStar (BMG Labtech, Mornington, VIC, Australia). TLR2 surface expression was determined by adding cultured cells to an antibody cocktail (TLR2; eBioscience, San Diego, CA, USA, and CD16 and CD45: BD Pharmingen, North Ryde, NSW, Australia) for 30 mins, and expression was corrected with matched isotypes. Cells were then assayed on FACSCanto II flow cytometer (BD Pharmingen) using BD FACSDiva 5 software. Data were exported from FACSDiva 5 software and analysed using FCAP Array Software (BD Pharmingen).

### 2.12. Data Analysis

Data were analysed using Stata 11 (Stata Corporation, College Station, TX, USA). Results are reported as mean (SD) or median (q1, q3) unless otherwise indicated. Analysis was performed using the two-sample Wilcoxon rank sum test, and the Kruskal-Wallis test was used for more than two groups. Fisher's exact test was used for categorical data. *In vitro* data were assessed using the Wilcoxon-signed rank test for paired data. Associations between data were determined using the Spearman rank correlation. Predictor variables were included in the multiple linear regressions if *P* < 0.1 in simple linear regression, and known confounders of age and gender were included in all models. Predicted variables were tested for colinearity using STATA's variance inflation factors postestimation. Flow cytometry relative median fluorescence intensity (RMFI) values were generated by dividing the MFI of the test antibody with that of the matched isotope control. All results were reported as significant, when *P* < 0.05.

## 3. Results

### 3.1. Study Population

One hundred and twenty adults participated in the study, comprising 69 adults with COPD aged >55 years and 51 healthy controls (29 aged ≥55 years older healthy controls and 22 aged <55 years of age, younger healthy controls). Demographic and inflammatory cell count data are shown in Tables 1 and 2, respectively.

The majority of participants with COPD had moderate airflow obstruction (*n* = 37, 54%), with 26 (38%) severe and 6 (9%) in the very severe category. Use of inhaled corticosteroids was common (87%) with a median (q1, q3) dose of 2000 (1000, 2000) *μ*g daily, and 57 (83%) were taking regular long-acting beta-agonists, while 46 (67%) were taking regular tiotropium bromide. In the 12 months prior to this study, 77% had visited their GP for their airways disease, 28% had been admitted to hospital, and 48% had received oral corticosteroids. Induced sputum samples of good quality were collected from 58 (92%) of participants, and analysis of cell differential and inflammatory mediators was carried out using only those participants with adequate sputum samples. Participants with COPD had an increased number of cells and cell viability, with increased proportion and number of neutrophils and eosinophils. There were also significantly lower proportions of sputum lymphocytes in patients with COPD compared with healthy controls (Table 2).

### 3.2. Effect of Age on Neutrophilic Inflammation and Innate Immune Mediator Expression

**Figure 1:**
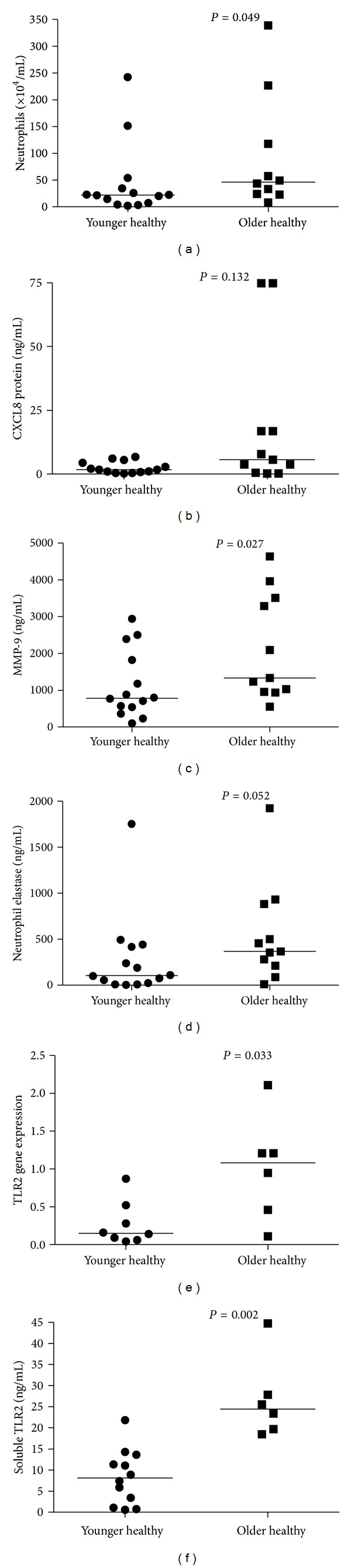
Analysis of the effect of age on markers of neutrophilic inflammation. This analysis used healthy never-smoking controls comparing younger (<55 years of age) with older (>55 years of age) controls. (a) Neutrophil number ×10^4^/mL, (b) CXCL8 protein ng/mL, (c) MMP-9 protein ng/mL, (d) NE protein ng/mL, (e) TLR2 gene expression, and (f) soluble TLR2 ng/mL.

To examine the effect of age we compared younger never-smoking controls with older never-smoking controls. There was a significant age-related increase in neutrophilic inflammation identified by increased sputum neutrophils (Figure 1(a)) and MMP-9 (Figure 1(c)). These increases were accompanied by enhanced TLR2 gene expression (Figure 1(e)) and soluble TLR2 protein levels (Figure 1(f)). There were no differences in CXCL8 (Figure 1(d)) or sputum TLR4 gene expression between younger controls (0.11 (0.0, 0.12)) and older controls (0.13 (0.10, 0.17), *P* = 0.172).

### 3.3. Effect of Smoking on Neutrophilic Inflammation and Innate Immune Mediators

To examine the effect of prior smoking we compared never-smoking controls without COPD (*n* = 27) to exsmoking controls without COPD (*n* = 15). 

The exsmoking controls had significant smoking exposure with 17 (1.5, 45) pack-years, but had a normal FEV_1_ (102% versus 107%, *P* = 0.385) and KCO% predicted (84% versus 82%, *P* = 0.605). They had quit smoking on average 25 years ago (SD 18, range 0.9–47.2) were older and more likely to be male (70% versus 39%, *P* = 0.033). 

**Figure 2:**
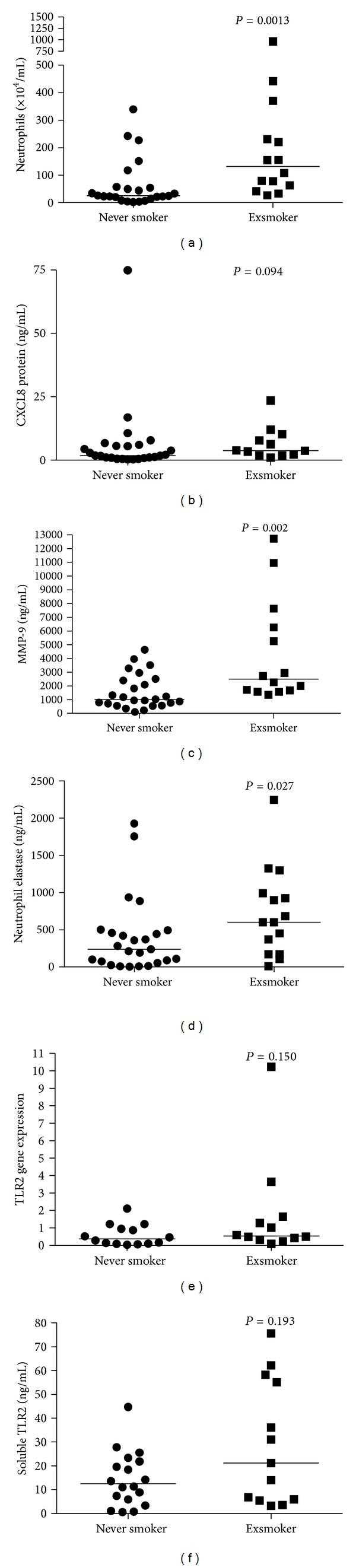
Analysis of the effect of smoking on markers of neutrophilic airway inflammation. This analysis used healthy controls, comparing never smokers with exsmokers. (a) Neutrophil number ×10^4^/mL, (b) CXCL8 protein ng/mL, (c) MMP-9 protein ng/mL, (d) NE protein ng/mL, (e) TLR2 gene expression, and (f) soluble TLR2 ng/mL.

Prior smoking was associated with elevated numbers of neutrophils (Figure 2(a)) and concentrations of proteases (MMP-9 and NE; Figures 2(c) and 2(d)). Although higher, the levels of CXCL8 (Figure 2(b)) in sputum supernatant from exsmokers were not statistically different (*P* = 0.094) compared with never smokers. There were no effects of smoking on sputum TLR2 gene expression (Figure 2(e)), soluble TLR2 levels (Figure 2(f)), or TLR4 gene expression (never smokers: 0.11 (0.0, 0.12) versus exsmokers: 0.13 (0.10, 0.17); *P* = 0.172).

### 3.4. Association between COPD and Neutrophilic Inflammation and Innate Immune Mediators

**Figure 3:**
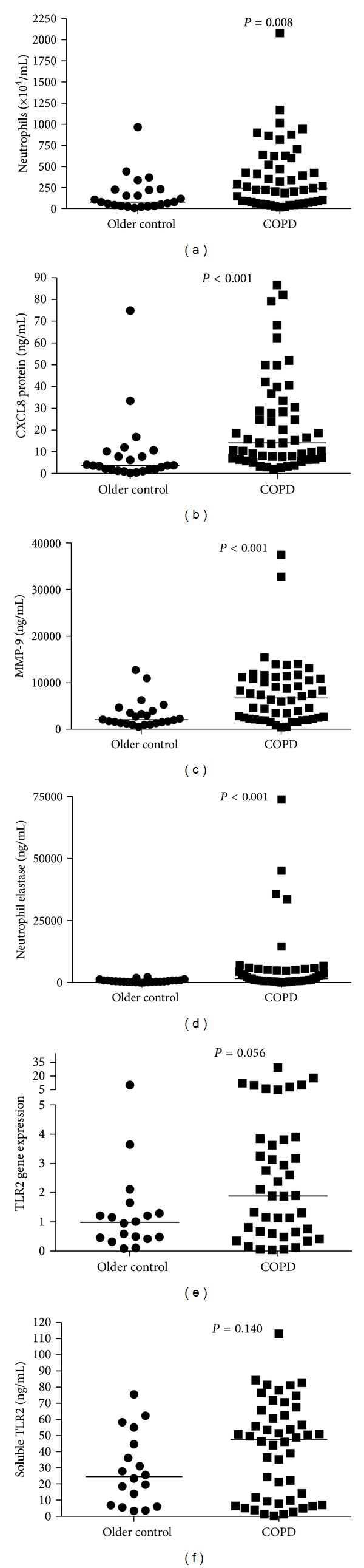
Analysis of the effect of the presence of airflow obstruction on markers of neutrophilic inflammation. This analysis compared older healthy controls with participants with COPD. (a) Neutrophil number ×10^4^/mL, (b) CXCL8 protein ng/mL, (c) MMP-9 protein ng/mL, (d) NE protein ng/mL, (e) TLR2 gene expression, and (f) soluble TLR2 ng/mL.

Next we examined the influence of COPD by comparing older healthy controls without COPD to those participants with COPD. We found increased neutrophils (Figure 3(a)) and CXCL8 (Figure 3(b)), MMP-9, and NE levels (Figures 3(c) and 3(d)) in the group with fixed airflow obstruction. While higher in patients with COPD, TLR2 gene expression (Figure 3(e)) and soluble TLR2 (Figure 3(f)) levels were not statistically elevated compared to healthy controls. There was no difference in sputum TLR4 gene expression between participants with COPD (0.15 (0.12, 0.20), compared with older controls (0.14 (0.10, 0.17, *P* = 0.203).

Both mRNA and soluble levels of TLR2 in patients with COPD were significantly associated with sputum levels of CXCL8 mRNA (*r* = 0.495, *P* < 0.001,  *N* = 41) and protein (*r* = 0.614, *P* < 0.001, *N* = 52), MMP-9 mRNA (*r* = 0.377, *P* = 0.0016, *N* = 41) and protein (*r* = 0.493, *P* < 0.001, *N* = 51), and NE mRNA (*r* = 0.518, *P* < 0.001, *N* = 39) and protein (*r* = 0.351,  *P* < 0.001,  *N* = 49). 

**Figure 4:**
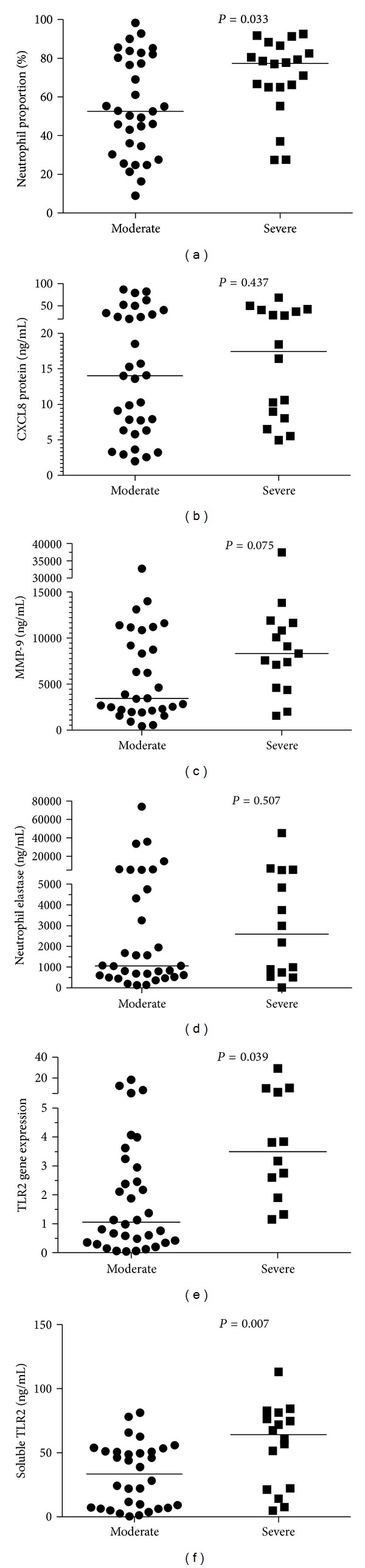
Analysis of the effect of the severity of airflow obstruction on markers of neutrophilic inflammation. This analysis compared participants with moderate and severe COPD. (a) Neutrophil number ×10^4^/mL, (b) CXCL8 protein ng/mL, (c) MMP-9 protein ng/mL, (d) NE protein ng/mL, (e) TLR2 gene expression, and (f) soluble TLR2 ng/mL.

We then compared clinical and inflammatory outcomes in patients with moderate and severe COPD. While similar in terms of age, gender, and smoking histories (Table 3), patients with severe COPD had a significantly higher proportion of sputum neutrophils (Figure 4(a)) as well as increased gene expression (Figure 4(e)) and levels of soluble TLR2 (Figure 4(f)).

### 3.5. TLR2 Gene Expression and Total MMP-9 Levels Are Independent Determinants of Neutrophilic Bronchitis

A multiple regression analysis was performed to further explore the determinants of neutrophilic bronchitis in all participants and to adjust for other determinants such as age, gender, and corticosteroid dose. Sputum neutrophils were significantly and independently associated with TLR2 gene expression and sputum MMP-9 levels (Table 4). Smoking history, age, inhaled corticosteroid dose, and airflow obstruction were not significantly correlated with sputum neutrophil proportion when adjusted for CXCL8, NE, and soluble TLR2 levels. The model was both highly significant and highly explanatory, with an adjusted *R* ^2^ of 59.08, *P* < 0.0001.

### 3.6. Peripheral Blood Cells Are Responsive to TLR2 Stimulation

**Figure 5:**
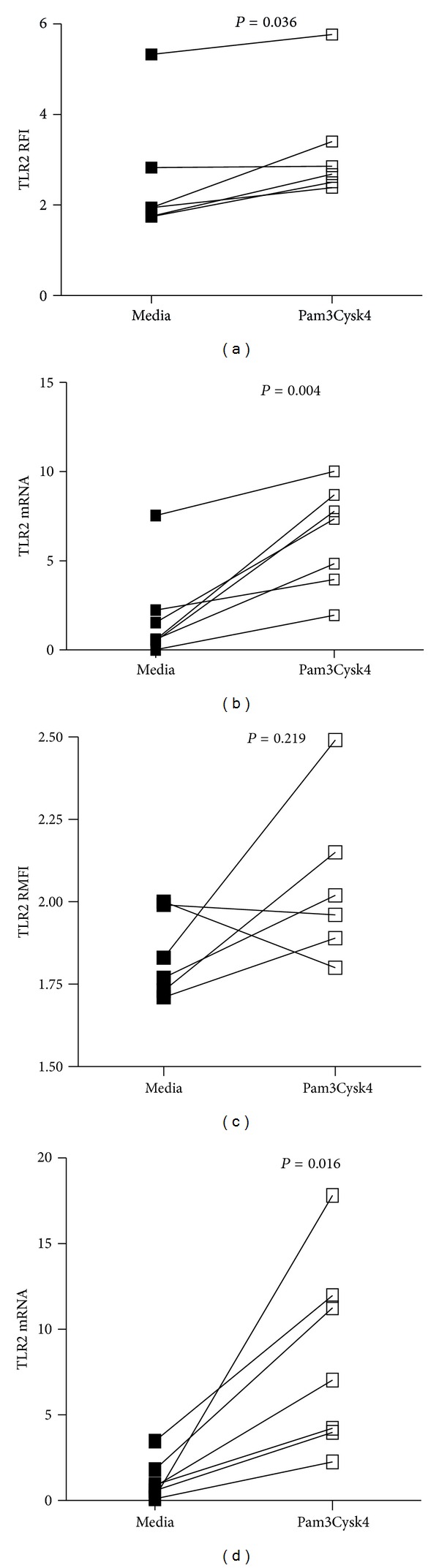
TLR2 surface and gene expression following TLR2 stimulation using Pam3CysK4: (a) mononuclear cells TLR2 surface expression, (b) mononuclear cells TLR2 gene expression, (c) granulocyte TLR2 surface expression, and (d) granulocyte TLR2 gene expression.

We assessed TLR2 activation in peripheral blood granulocytes and mononuclear cells in 7 participants with COPD with a median (q1, q3) age of 65 (63, 67) years, 3 were male, and 4 were exsmokers. The median (q1, q3) FEV_1_% predicted was 51.4 (40.8, 60.5), and FEV_1_/FVC was 61.9 (42.9, 68.0). Following TLR2 stimulation of PBMCs from patients with COPD, both surface and gene expression of TLR2 were increased (Figures 5(a) and 5(b)). There was a significant increase in TLR2 gene expression but not surface expression on peripheral granulocytes (Figures 5(c) and 5(d)). 

### 3.7. Neutrophil Proteases and Innate Inflammatory Cytokines Are Released Following TLR2 Stimulation of COPD Peripheral Blood Cells 

**Figure 6:**
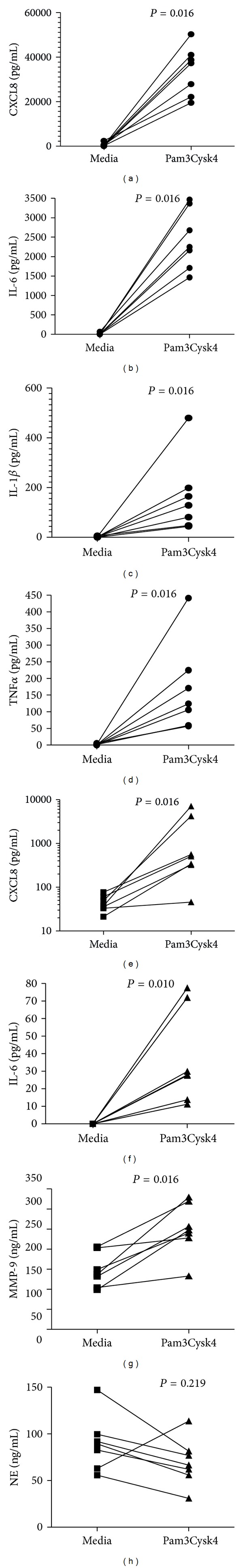
Release of inflammatory mediators from mononuclear cells and granulocytes isolated from patients with COPD. (a) CXCL8 release from mononuclear cells, (b) IL-6 release from mononuclear cells, (c) IL-1*β* release from mononuclear cells, (d) TNF-*α*  release from mononuclear cells, (e) CXCL8 release from granulocytes, (f) IL-6 release from granulocytes, (g) MMP-9 release from granulocytes, and (h) NE release from granulocytes.

Peripheral blood cells were stimulated with Pam3CysK4 and supernatant examined for release of inflammatory cytokines and proteases. TLR2 stimulation resulted in increased release of CXCL8, IL-6, IL-1*β*, and TNF-*α*  from peripheral blood mononuclear cells (Figures 6(a)–6(d)) and CXCL8, IL-6, and MMP-9 from granulocytes (Figures 6(e)–6(g)). Release of NE was not altered with Pam3CysK4 stimulation (Figure 6(h)). 

MMP-9 and NE were only assessed in granulocyte cultures' culture supernatant only as they are predominantly neutrophil proteases.

## 4. Discussion

This study evaluated the determinants of the persistent neutrophilic bronchitis, that is a characteristic of COPD, and the role of ageing and smoking, and COPD severity. Elevated TLR2 levels were associated with ageing and more severe COPD. Sputum neutrophils and total MMP-9 levels increased with age, prior smoking, and the presence of fixed airflow obstruction. Gene expression for TLR2 and supernatant MMP-9 levels were significant independent contributors to the proportion of sputum neutrophils after adjustment for age, prior smoking, and the presence of airflow obstruction. The TLR2 receptor was functionally active in COPD with stimulation of peripheral blood granulocytes and mononuclear cells leading to the release of key neutrophil and innate immune system mediators including MMP-9. These results implicate a significant role for the innate immune system in neutrophilic bronchitis in COPD and suggest activation of TLR2 and subsequent release of MMP-9 may be critical points in the regulation of neutrophilic bronchitis in COPD.

Ageing, smoking, and airflow obstruction have each been linked to airway neutrophilia. A number of studies have reported similar or increased circulating neutrophil numbers in the elderly [16], but few have examined airway neutrophils and associated inflammation in older people. Thomas et al. reported a significant positive relationship between neutrophils and age after examining sputum neutrophils in >60 healthy nonsmoking controls, with older controls having a neutrophil proportion of 68.5% compared with younger healthy controls [17]. We extend these findings by demonstrating age-related increases in TLR2 gene expression, soluble TLR2 levels, and MMP-9 levels. Age has been shown to have no effect on the expression of TLR2 and -4 on peripheral blood neutrophils using flow cytometry [18]. In our examination of induced sputum, we found that TLR2 but not TLR4 gene expression was increased in older healthy controls compared with younger healthy controls, indicating that TLR2 expression increases with age. We also observed higher levels of soluble TLR2 in older nonsmoking controls compared with younger nonsmoking controls. These changes could represent airway centric changes in innate immune receptor expression and function with age, which is supported by a recent animal study showing increased airway macrophage TLR2 expression with advanced age [19].

Prior smoking was associated with increased airway neutrophils and release of MMP-9 and NE. These data extend previous reports of associations between smoking and reduced lung function, neutrophilia and increased levels of CXCL8 in sputum supernatants from younger controls, [20] and elevated levels of MMP-9 in current smokers compared with nonsmokers [21]. We observed similar effects in exsmokers suggesting that cigarette smoking may irreversibly exaggerate innate immune responses and lead to low grade neutrophilic inflammation, prior to the clinical expression of airway disease. Indeed, when active smokers cease smoking, MMP-9 levels remain unaltered [22]. Alterations in MMP-9 may serve as an early indicator of smoking-related airway damage due to elevation in both asymptomatic exsmokers (shown here) and current smokers [23], and further work is required to determine its suitability as a biomarker of smoking-related airway disease. 

Neutrophilic bronchitis is also a feature of other chronic airway diseases such as bronchiectasis [5], allergic bronchopulmonary aspergillosis [24], and the neutrophilic subtype of asthma [25]. In these diseases there are increased levels of mediators of neutrophilic inflammation (CXCL8), neutrophil elastase (NE), and matrix metalloproteinase- (MMP-) 9 and strong associations observed between the expression of these mediators and elevated numbers of sputum neutrophils. This suggests there may be common mechanisms contributing to persistent neutrophilia in airway disease and COPD [26–28], and in this study we have observed similar increases in mediators of neutrophilic inflammation and associations between mediators and sputum neutrophil proportion. In cell culture systems, neutrophils release MMP-9 in the presence of CXCL8, and NE can induce CXCL8, which indicates that neutrophils can perpetuate their own accumulation and activation [29, 30].

A likely explanation of this specific enhancement of neutrophilic inflammation may lie in the cumulative effect of the exposures experienced by participants. These exposures included not only tobacco smoke, but also likely other pollutants or past infections all of which can induce epigenetic alterations, and these possibilities require further investigations [31]. The hypothesis of  “multiple inflammatory hits” initially proposed by Pavord et al., in severe refractory asthma [32], may explain the changes in disease parameters observed in this study, with those with COPD having more inflammatory “hits” than exsmoking or nonsmoking controls.

The COPD population in this study was primarily treated with maintenance inhaled corticosteroids which is common and recommended in COPD. A small proportion of those with COPD showed a response to bronchodilator on the day of assessment, and around 25% had a sputum eosinophil proportion of more than 3% which suggests further management with inhaled corticosteroids or an assessment of therapy may be considered. While these are characteristics of patients with asthma, asthma is also a significant risk factor for the development of COPD; therefore this is not a surprising result. Approximately half of the COPD population studied were atopic. In a recent study the presence of atopy in COPD was associated with increased respiratory symptoms and more likely to have a resolution of symptoms on treatment with budesonide [33], and the presence of atopy is considered to be a “minor” criteria for the definition of asthma/COPD overlap [34].

Innate immune responses are rapidly activated by pathogen-associated molecular patterns, which are highly conserved microbial factors. They are recognised by pattern recognition receptors such as toll-like receptors (TLRs), CD14, and collectins [35]. TLR activation triggers signaling cascades that lead to the activation and nuclear translocation of NF-*κ*B resulting in proinflammatory cytokine responses including TNF-*α*, IL-8, and IL-1*β* [36, 37].

Stimulation of TLR2 induced increased TLR2 gene expression in peripheral blood granulocytes and increased release of the neutrophil chemoattractant CXCL8 and the neutrophil protease MMP-9, while stimulation of peripheral blood monocytic cells leads to increased gene and surface expression of TLR2 as well as the release of CXCL8 and innate cytokines TNF-*α*  and IL-1*β*. It is a limitation to this study that we were not able to show TLR2 activation of airway cells. We have previously reported that sputum cells are refractory to stimulation with LPS which may be due to the cells already being maximally activated, whether this occurs while in the airway or as a result of the sputum induction process which requires further study [38].

We extend the findings of Von Scheele et al. [8] to show that soluble levels of TLR2 are further elevated in severe compared with moderate COPD and that, in participants with COPD, soluble TLR2 levels are closely associated with sputum levels of CXCL8, which is the key neutrophil chemoattractant. The levels of soluble TLR2 correlated with increased levels of MMP-9 and NE, and it is possible that these proteases may cleave off TLR2 from cells increasing the levels of the soluble form and influence their ability to respond to agonists of the receptor. Stimulation with the TLR2 agonist Pam3CysK4 has been shown to induce MMP-9 production in other cell types such as THP-1 cells and keratinocytes [39]; our work extends these observations to show that stimulation of granulocytes with Pam3CysK4 also induces projection of MMP-9 and suggests the potential of TLR2 to influence tissue remodeling and repair in the airways. Corticosteroids have also been shown to enhance the TLR2 response in airway epithelial cells [40–42], and thus it is important that further research is undertaken to elucidate the effect of steroids on TLR2 expression and responsiveness in COPD.

Animal studies also support key roles for TLR2 as a determinant of airway neutrophilia. TLR2 (but not TLR4) expression is increased on lung macrophages in a mouse model of infection-induced neutrophilic allergic airway disease [43]. In a model of organic dust-induced airway inflammation, TLR2 knockout mice had significantly lower airway neutrophils, IL-6, TNF-*α*, and CXCL-1 (mouse homolog of CXCL8) compared with wild-type controls suggesting that airway inflammation is dependent on TLR2 [44]. This is the same pattern of cytokines we observed following TLR2 stimulation in peripheral blood cells from patients with COPD and in induced sputum samples from patients with COPD. 

In conclusion, we have shown that airway neutrophilia and MMP-9 are enhanced with age, smoking, and the presence of COPD. Sputum TLR2 gene expression was significantly increased with age but not prior smoking; however it was enhanced in COPD and increased with COPD severity. TLR2 gene expression and MMP-9 levels were significant independent predictors of neutrophils in sputum, and the receptor was shown to be functional and could be activated to induce the release of MMP-9 and CXCL8 from granulocytes as well as innate cytokines IL-1*β* and TNF-*α*  from monocytes. These results extend our understanding of the role of the innate immune response in inducing neutrophilia in airways disease and provide new insights for the development of effective therapies. 

## Figures and Tables

**Table 1:** Clinical characteristics for all participants. Data are median (q1, q3) unless indicated.

	COPD	Older healthy control	Younger healthy control	*P*
*n*	69	29	22	
Age, years mean (SD)	70.0 (7.3)	65.9 (6.8)*	32.3 (10.0)^∗†^	<0.001
Sex, *n* (%) males	34 (49)	15 (52)	6 (27)	0.157
Smoker, never/ex	17/52	13/16	20/2^∗†^	<0.001
Pack years	34.8 (18.3, 56.3)	18.5 (2.1, 41.9)	2.9, 4.5	0.056
Years since ceased smoking (exsmokers), mean (SD)	19.8 (13.5)	24.4 (17.5)	17.3 (9.5)	0.515
Atopic, *n* (%)	36 (52)	8 (28)	12 (55)	0.057
More than 2 chest infections reported in last year, *n* (%)	18 (29)	0 (0)	0 (0)*	0.005
SF-36 physical	33.2 (27.1, 42.6)	53.1 (50.1, 56.2)*	54.8 (52.9, 57.8)*	<0.001
SF-36 mental	52.0 (38.1, 59.4)	57.5 (55.5, 60.5)*	55.6 (54.4, 57.0)*	<0.001
Bronchodilator response %,	9.6 (4.1, 15.3)	4.5 (2.4, 6.9)*	4.2 (2.6, 5.9)*	<0.001
Bronchodilator response, *n* (%)	11 (16)	1 (3)	1 (5)	0.127
FEV_1_% predicted, mean (SD)	52.1 (15.4)	102.8 (16.4)*	108.7 (12.3)*	<0.001
FEV_1_/FVC%, mean (SD)	52.2 (11.5)	77.5 (4.1)*	84.8 (5.7)*	<0.001
KCO% predicted, mean (SD)	53.6 (23.8)	81.1 (10.8)*	86.7 (9.9)^∗†^	<0.001

*P < 0.05 versus COPD; ^†^ *P* < 0.05 versus older healthy control.

SF-36: a short form health survey of 36 questions generic measure of quality of life.

**Table 2:** Airway inflammation data. All data are median (q1, q3) unless stated.

	COPD	Older healthy control	Younger healthy control	*P*
Number of patients where sputum was induced, *n* (%)	64 (93)	28 (97)	22 (100)	0.529
Number of patients with adequate sputum used in analysis, *n* (%)	58 (90)	26 (93)	16 (73)	0.096
Total cell count, ×10^6^/mL^a^	4.3 (1.7, 6.7), *n* = 51	2.3 (1.4, 3.8), *n* = 23	1.7 (1.1, 3.2)*, *n* = 15	0.005
Viability, %	81 (67, 93)	79 (67, 89)	67 (59, 76)*	0.015
Neutrophils, %	65.0 (43.0, 82.5)	39.6 (27.8, 60.0)*	10.8 (7.8, 28.4)^ ∗†^	<0.001
Neutrophils, 10^4^/mL	244 (78.4, 599)	79.2 (33.1, 227)*	22.2 (6.8, 41.6)^ ∗†^	<0.001
Eosinophils, %	1.0 (0.3, 2.8)	0.3 (0.0, 0.5)*	0.1 (0.0, 0.4)*	<0.001
Eosinophils, ×10^4^/mL	5.1 (0.8, 16.7)	0.6 (0.0, 1.6)*	0.0 (0.0, 0.6)*	<0.001
Macrophages, %	28.4 (12.5, 47.5)	49.1 (34.8, 68.8)*	81.3 (64.6, 89.5)*	<0.001
Macrophages, ×10^4^/mL	78.0 (48.6, 139.5)	117.0 (69.1, 161.3)	137.3 (57.5, 194.1)	0.144
Lymphocytes, %	0.3 (0.0, 0.8)	0.8 (0.0, 1.8)*	1.0 (0.0, 1.9)	0.019
Lymphocytes, ×10^4^/mL	0.4 (0.0, 1.7)	1.8 (0.0, 4.2)	1.4 (0.0, 5.1)	0.087
Columnar epithelial cells, %	1.6 (0.5, 2.5)	1.2 (0.3, 2.3)	2.9 (0.4, 6.5)	0.557
Columnar epithelial cells, ×10^4^/mL	5.6 (1.6, 10.4)	2.3 (0.5, 9.8)	4.5 (0.2, 21.1)	0.461
Squamous cells, %	3.5 (1.2, 8.9)	3.6 (2.2, 7.6)	6.7 (3.4, 13.4)	0.135

*P < 0.05 versus COPD; ^†^ *P* < 0.05 versus older healthy control.

^a^When sputum volume was small, a cell smear was prepared and total cell count and viability data were not collected.

**Table 3:** Clinical and inflammatory outcomes according to the severity of airflow obstruction in patients with COPD. Data are median (q1, q3) unless indicated.

	Moderate COPD	Severe COPD	*P*
*n*	34	20	
Age, mean (SD)	70.4 (7.3)	69.9 (7.9)	0.844
Sex, male/female	16/28	9/11	0.555
Smoker, never/ex	12/22	3/17	0.096
Pack years	32.3 (17, 46.6)	37.3 (19.5, 53.8)	0.444
FEV_1_% predicted	65.3 (57.9, 69.8)	43.8 (38.2, 46.8)	<0.001
ICS dose	2000 (1000, 2000)	2000 (2000, 2000)	0.183
TLR4 mRNA	0.15 (0.12, 0.18), *N* = 26	0.16 (0.11, 0.22), *N* = 12	0.648

**Table 4:** Multivariate linear regression outcomes to determine predictors of sputum neutrophil proportion.

Variable	Coefficient	SE	*P*	95% confidence interval
Age, years	0.068	0.232	0.771	−0.400 to 0.537
Sex (male/female)	8.968	6.596	0.181	−4.342 to 22.278
Smoker (ex/never)	2.988	6.994	0.671	−11.123 to 17.103
FEV_1_/FVC, %	−0.130	0.239	0.589	−0.613 to 0.353
Soluble TLR2 ng/mL	−0.048	0.137	0.729	−0.324 to 0.228
**Log TLR2 gene expression**	**16.244**	**4.793**	**0.002**	**6.570 to 25.917**
Log TLR4 gene expression	12.405	9.104	0.180	−5.968 to 30.778
**Log MMP-9 protein**	**34.523**	**10.253**	**0.002**	**13.831 to 55.215**
Log NE	2.122	5.566	0.705	−9.110 to 13.354
Log CXCL8	−3.969	8.292	0.635	−20.703 to 12.765
Dose-inhaled corticosteroids, *μ*g daily	−0.003	0.003	0.369	−0.009 to 0.004
Constant	−69.667	46.503	0.142	−163.514 to 24.181
